# Machine learning-based prediction model of acute kidney injury in patients with acute respiratory distress syndrome

**DOI:** 10.1186/s12890-023-02663-6

**Published:** 2023-10-03

**Authors:** Shuxing Wei, Yongsheng Zhang, Hongmeng Dong, Ying Chen, Xiya Wang, Xiaomei Zhu, Guang Zhang, Shubin Guo

**Affiliations:** 1 Emergency Medicine Clinical Research Center, Beijing Key Laboratory of Cardiopulmonary Cerebral Resuscitation, Beijing Chaoyang Hospital Affiliated to Capital Medical University, Beijing, 100020 China; 2 Department of Health Management, Shandong Engineering Laboratory of Health Management, Institute of Health Management, the First Affiliated Hospital of Shandong First Medical University & Shandong Provincial Qianfoshan Hospital, Jinan, 250014 China

**Keywords:** Acute respiratory distress syndrome, Acute kidney injury, Machine learning

## Abstract

**Background:**

Acute kidney injury (AKI) can make cases of acute respiratory distress syndrome (ARDS) more complex, and the combination of the two can significantly worsen the prognosis. Our objective is to utilize machine learning (ML) techniques to construct models that can promptly identify the risk of AKI in ARDS patients.

**Method:**

We obtained data regarding ARDS patients from the Medical Information Mart for Intensive Care III (MIMIC-III) and MIMIC-IV databases. Within the MIMIC-III dataset, we developed 11 ML prediction models. By evaluating various metrics, we visualized the importance of its features using Shapley additive explanations (SHAP). We then created a more concise model using fewer variables, and optimized it using hyperparameter optimization (HPO). The model was validated using the MIMIC-IV dataset.

**Result:**

A total of 928 ARDS patients without AKI were included in the analysis from the MIMIC-III dataset, and among them, 179 (19.3%) developed AKI after admission to the intensive care unit (ICU). In the MIMIC-IV dataset, there were 653 ARDS patients included in the analysis, and among them, 237 (36.3%) developed AKI. A total of 43 features were used to build the model. Among all models, eXtreme gradient boosting (XGBoost) performed the best. We used the top 10 features to build a compact model with an area under the curve (AUC) of 0.850, which improved to an AUC of 0.865 after the HPO. In extra validation set, XGBoost_HPO achieved an AUC of 0.854. The accuracy, sensitivity, specificity, positive prediction value (PPV), negative prediction value (NPV), and F1 score of the XGBoost_HPO model on the test set are 0.865, 0.813, 0.877, 0.578, 0.957 and 0.675, respectively. On extra validation set, they are 0.724, 0.789, 0.688, 0.590, 0.851, and 0.675, respectively.

**Conclusion:**

ML algorithms, especially XGBoost, are reliable for predicting AKI in ARDS patients. The compact model maintains excellent predictive ability, and the web-based calculator improves clinical convenience. This provides valuable guidance in identifying AKI in ARDS, leading to improved patient outcomes.

**Supplementary Information:**

The online version contains supplementary material available at 10.1186/s12890-023-02663-6.

## Background

Acute respiratory distress syndrome (ARDS) is a sudden-onset respiratory illness that is identified by the presence of opacities in the chest radiographs of both lungs [1]. ARDS is a severe respiratory condition that poses a significant risk to patients, with high morbidity and mortality rates. A comprehensive observational study carried out across 50 countries found that approximately 10.4% of intensive care unit (ICU) admissions are due to ARDS. Unfortunately, the in-hospital mortality rate for patients with ARDS exceeds 30%, making it a critical medical emergency that requires prompt and effective management [2]. A study has revealed that approximately 33% of patients who receive mechanical ventilation in the ICU are susceptible to developing ARDS. Individuals who are at risk for ARDS frequently experience lung complications, and their clinical outcomes are often poorer than those who are not at risk for ARDS [3]. Acute kidney injury (AKI) is a prevalent complication that may occur in patients with ARDS, and it is typically linked with a bleak prognosis. Studies have demonstrated that individuals with ARDS who develop AKI usually need extended periods of mechanical ventilation compared to those who do not experience AKI, and they also tend to have lengthier hospital stays and an increased risk of mortality [4, 5]. Studies have shown that AKI is a common complication in patients with ARDS and is associated with a significantly higher mortality rate. The ARDSnet trial found that approximately 24% of participants with ARDS developed AKI, and those with AKI had a much higher 180-day mortality rate compared to those without AKI (58% versus 28%) [6]. Similarly, a multi-center study from France showed that AKI occurred in 44.3% of ARDS patients and was associated with higher mortality rates compared to those without AKI (42.3% versus 20.2%) [7]. These findings highlight the significance of identifying and treating AKI promptly in patients with ARDS, and emphasize the necessity of monitoring kidney function closely in this patient cohort. By implementing successful measures to prevent and manage AKI in ARDS patients, outcomes can potentially be enhanced, and the risk of mortality minimized.

Currently, there is limited research for AKI occurrence in ARDS patients. One study demonstrated that red cell volume distribution width (RDW) is an independent predictor of AKI in ARDS patients, with an area under the curve (AUC) of 0.687 [8]. Another study utilized data from Medical Information Mart for Intensive Care III (MIMIC-III) to construct a machine learning (ML) model for AKI in sepsis-related ARDS patients, with the eXtreme gradient boosting (XGBoost) model showing the best performance and an AUC of 0.859. However, this model was not validated [9]. And this model is only applicable specifically to ARDS caused by sepsis.

ML is a sophisticated modeling technique that has emerged as a game-changer in recent years, outperforming traditional risk models such as logistic regression analysis [10]. ML’s key advantage lies in its ability to automatically recognize complex relationships between variables and response values from vast amounts of data. This capability results in improved performance by identifying crucial predictive variables and making more accurate predictions [11]. ML algorithms can handle intricate and high-dimensional data that is often encountered in modern scientific and medical research, setting it apart from traditional methods [12]. As a result, ML has become an essential tool for analyzing big data in a wide range of fields, including healthcare, finance, and engineering. By uncovering hidden patterns and relationships in data, ML has the potential to revolutionize scientific research and lead to more effective and efficient decision-making, ultimately driving innovation and progress in many areas of society [13–15].

The primary objective of this study is to leverage ML to identify the biological and clinical factors that predict the occurrence of AKI in ARDS patients. By constructing a robust AKI prediction model and validating it thoroughly, we aim to detect AKI in ARDS patients, which can lead to better patient outcomes and provide new insights into prevention and treatment strategies for patients with ARDS.

## Methods

### Data source

Using Structured Query Language, data was extracted from a single-center public database known as the MIMIC-III and MIMIC-IV databases [16]. MIMIC-III is a comprehensive clinical dataset that contains information on all patients who were admitted to the ICU at Beth Israel Deaconess Medical Center in Boston, Massachusetts between 2001 and 2012. MIMIC-IV database is the latest update to MIMIC-III database [17]. The databases provide detailed information on various aspects of patient care, including demographic features, vital sign monitoring, laboratory and microbiological tests, intake and output observations, medication therapies, hospitalization duration, survival data, and discharge or death records. We obtained institutional review board approval to ensure the protection of human research participants, and we obtained a certificate (Certification Number: 47,937,607) that enabled us to access the database. We selected patients in MIMIC-IV who were hospitalized after 2014 to avoid overlapping with MIMIC-III.

### Participants

Our study enrolled patients who met the following eligibility criteria: they were 16 years of age or older, had been hospitalized in the ICU for more than 24 h, and were diagnosed with ARDS according to the Berlin criteria [18]within 24 h of admission to the ICU. We only included data from the first admission for patients who were admitted to the ICU multiple times. Patients who had an initial partial arterial oxygen pressure (PaO_2_)/ fraction of inspiration O_2_ (FiO_2_) ratio between 201 and 300 mmHg and were given invasive or noninvasive ventilation via a tight mask and positive end expiratory pressure (PEEP) of at least 5 cm H_2_O were categorized as having mild ARDS according to the Berlin criteria. Moderate ARDS was identified as a PaO_2_/FiO_2_ ratio ranging from 101 to 200 mmHg, while severe ARDS was classified as a PaO_2_/FiO_2_ ratio of 100 mmHg or less. Furthermore, we identified patients who exhibited bilateral chest CT scan infiltrates that met the Berlin criteria. We utilized the ICD-9 code to diagnose cases of AKI and excluded patients who had chronic kidney disease (CKD) or end-stage renal disease (ESRD), or whose creatinine levels were ≥ 4 mg/dL upon admission to the study.

### Data

Features with missing values exceeding 20% were discarded, and multiple imputation by chained equations was used to impute missing values in the remaining feature space. The study utilized the following information: (1) demographic characteristics such as sex, age, and body mass index (BMI); (2) comorbidities, including urinary tract infection (UTI), diabetes, and sepsis; (3) vital signs, including respiratory rate (RR), heart rate (HR), temperature, oxygen saturation (SpO_2_), systolic blood pressure (SBP), diastolic blood pressure (DBP), and mean arterial pressure (MAP); (4) laboratory parameters, such as base excess (BE), blood urea nitrogen (BUN), albumin, calcium, chloride, potassium, sodium, creatinine, glucose, actual bicarbonate radical (ABC), hematocrit, hemoglobin, PH, lactate, phosphate, PaO_2_, partial pressure of carbon dioxide (PCO_2_), red blood cell (RBC) and white blood cell (WBC) counts, alanine aminotransferase (ALT), aspartate aminotransferase (AST), total bilirubin (TBIL), RDW, international normalized ratio (INR), partial thromboplastin time (PTT), prothrombin time (PT), and urine output (UO). A total of 46 variables were included in the analysis, which included the patient’s PEEP value and ARDS classification. For variables that were measured multiple times, we only included the first measurement in the analysis.

### Statistical analysis

Categorical variables were represented as number and percentage and were compared using the Chi-square test. The Kolmogorov-Smirnov test was used to assess the normal distribution of continuous variables. If the data exhibited a normal distribution, T-tests were performed, utilizing mean and standard deviation as descriptive statistics for the variables. Conversely, for non-normally distributed variables, the Wilcoxon rank-sum test was employed, and descriptive statistics (median and extremums) were used to characterize the variables. Subsequently, the data in MIMIC-III were randomly divided into a training set and a testing set in a 8:2 ratio. We utilized the synthetic minority over-sampling technique (SMOTE) algorithm in the training set to enhance the predictive performance of the ML models for minority classes and improve the handling of imbalanced datasets. This study established 11 ML models, including logistic regression, K-nearest neighbor (KNN), decision tree, random forest, support vector machine (SVM), XGBoost, adaptive boosting (AdaBoost), gradient boosting decision tree (GBDT), multi-layer perception (MLP), light gradients boosting machine (LightGBM), and category boosting (CatBoost). In addition, the established model was compared with the sequential organ failure assessment (SOFA) score. The models were evaluated based on the testing set, using AUC, accuracy, sensitivity, specificity, positive prediction value (PPV), negative prediction value (NPV), and F1 score, and then the best model was selected. To enhance the interpretability of our top-performing model, we employed the shapley additive explanations (SHAP) approach. We visualized the impact of the model’s features using a SHAP summary plot, which allowed us to understand how each feature contributed to the overall prediction. To facilitate clinical use, we simplified the complex model into a compact model. Subsequently, the hyperparameter optimization (HPO) was conducted to improve the performance of the compact model. To optimize our model, we used Optuna version 2.10., which is an open-source hyperparameter optimization framework that can automatically choose the best hyperparameters, specifically designed for ML. We validated the the ML models on the validation set (MIMIC-IV database). Next, we developed a web-based interactive ML program for the daily use of the optimal prediction model. In addition, we use the calibration curve to evaluate the relationship between the predicted values of the model and the actual observed values, as well as the uncertainty of the model predictions. All analyses were performed using Python (v.3.9.12) and R (v.4.2.0, R Foundation for Statistical Computing). *P* values less than 0.05 were considered statistically significant.

## Results

### Baseline characteristics

**Fig. 1:**
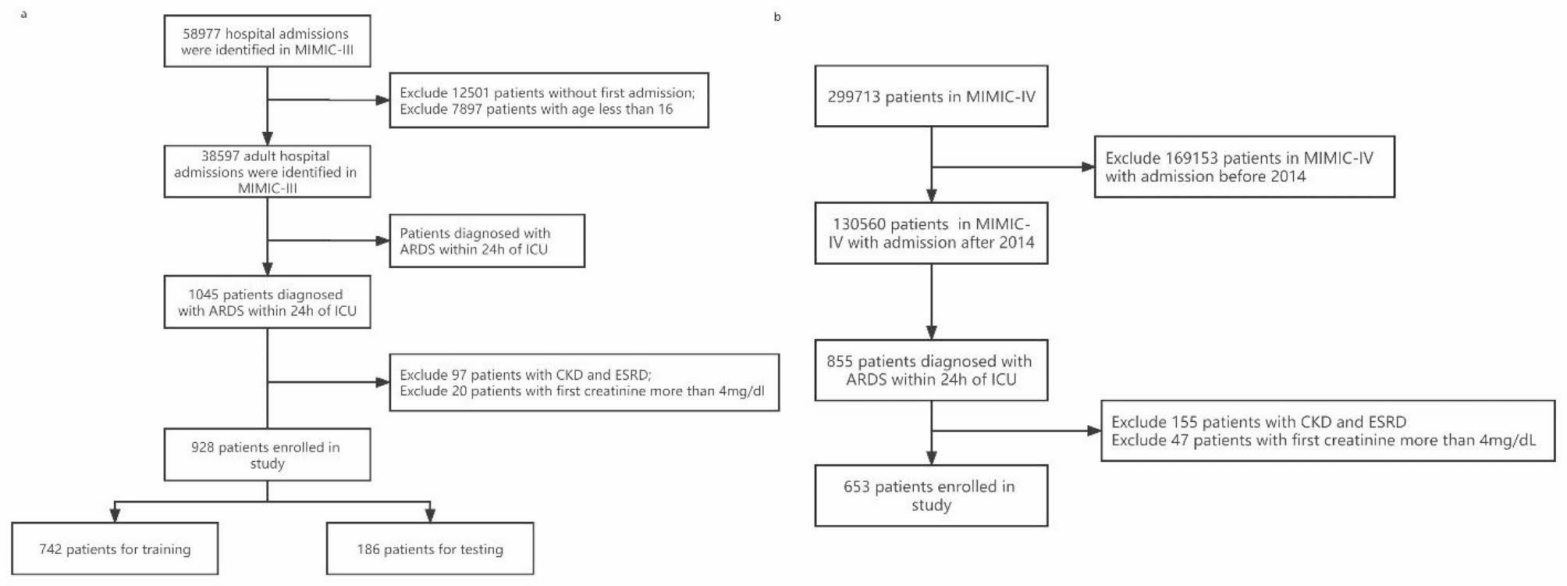
**a** The flowchart of ARDS patients in MIMIC-III. **b** The flowchart of ARDS patients in MIMIC-IV. MIMIC, Medical Information Mort for Intensive Care; ARDS, acute respiratory distress syndrome; ICU, intensive care unit; CKD, chronic kidney disease; ESRD, end-stage renal disease

According to the inclusion criteria outlined in Fig. 1, a total of 928 ARDS patients were included in the MIMIC-III database. Additionally, the MIMIC-VI database included a total of 653 patients. Out of the 928 patients, 563 (60.7%) were male and 179 (19.2%) developed AKI during their hospital stay. Of those who developed AKI, 91 cases (50.8%) were diagnosed with severe ARDS, which was higher than the non-AKI group (291 cases, 38.9%). Our study included 42 predictive variables, and we found that ARDS patients with AKI had a higher likelihood of having other comorbidities such as sepsis [78 (43.6%) vs. 50 (6.7%), P < 0.001], diabetes [59 (33.0%) vs. 166 (22.2%), P = 0.00337], and UTI [32 (17.9%) vs. 68 (9.1%), P = 0.00105]. Additionally, compared to the non-AKI group, the AKI group had higher mean PEEP values (6.75 vs. 5.62, P < 0.001) and higher first admission creatinine values (1.49 vs. 0.851, P < 0.001). Comparisons between the non-AKI and AKI groups are shown in Table 1.

**Table 1:** Baseline characteristics of ARDS patients in MIMIC-III

	Non-AKI (N = 749)	AKI (N = 179)	P-value
**Gender**			0.315
Male	448 (59.8%)	115 (64.2%)	
Female	301 (40.2%)	64 (35.8%)	
**Age**			0.259
Under 50	187 (25.0%)	38 (21.2%)	
Between 50–70	355 (47.4%)	97 (54.2%)	
Above 70	207 (27.6%)	44 (24.6%)	
**ARDS**			0.012
Mild-ARDS	117 (15.6%)	25 (14.0%)	
Severe-ARDS	291 (38.9%)	91 (50.8%)	
Moderate-ARDS	341 (45.5%)	63 (35.2%)	
BMI*	28.4 [9.86, 115]	28.3 [10.8, 91.6]	0.907
BE*	1.00 [-33.0, 16.0]	-1.00 [-34.0, 12.0]	< 0.001
BUN*	16.0 [3.00, 76.0]	28.0 [7.00, 114]	< 0.001
**Calcium**	8.40 (0.926)	8.17 (0.965)	0.00386
**Chloride**	105 (6.05)	104 (7.04)	0.0823
Creatinine*	0.800 [0.200, 2.50]	1.30 [0.300, 3.80]	< 0.001
**DBP**	65.0 (14.9)	63.3 (15.7)	0.19
Glucose*	121 [39.0, 508]	126 [40.0, 560]	0.0668
**HCO**_**3**_	25.7 (4.34)	24.0 (5.32)	< 0.001
**Hematocrit**	32.7 (6.22)	32.5 (6.95)	0.688
**Hemoglobin**	11.0 (2.15)	10.8 (2.32)	0.23
**HR**	87.3 (16.8)	95.1 (19.0)	< 0.001
**Potassium**	4.10 (0.567)	4.22 (0.684)	0.0324
Lactate*	1.50 [0.400, 13.5]	2.20 [0.700, 12.7]	< 0.001
**MAP**	82.3 (15.7)	78.5 (16.9)	0.00554
**Sodium**	139 (4.48)	139 (6.59)	0.705
**PCO**_**2**_ *	42.0 [16.0, 119]	42.0 [19.0, 96.0]	0.327
**PH**	7.38 (0.0969)	7.34 (0.124)	< 0.001
Phosphate*	3.30 [1.20, 9.20]	3.70 [1.30, 12.4]	< 0.001
Platelets*	212 [5.00, 871]	214 [16.0, 796]	0.228
**PO**_**2**_ *	233 [24.0, 567]	107 [24.0, 607]	< 0.001
**RBC**	3.69 (0.741)	3.59 (0.813)	0.144
RR*	0 [0, 45.0]	0 [0, 43.0]	0.245
**SBP**	121 (23.2)	118 (24.9)	0.234
**SpO**_**2**_	98.1 (4.24)	96.5 (4.01)	< 0.001
Temperature*	36.8 (0.852)	36.9 (1.15)	0.22
WBC*	10.6 [0.300, 46.8]	11.7 [0.400, 50.7]	0.00905
UO*	1650 [20.0, 6090]	1180 [30.0, 5980]	0.00153
**Albumin**	3.41 (0.712)	2.98 (0.759)	< 0.001
ALT*	25.0 [3.00, 1770]	32.0 [6.00, 2550]	0.00879
AST*	31.0 [7.00, 1900]	51.0 [10.0, 5570]	0.00404
TBIL*	0.500 [0.100, 37.5]	0.800 [0.100, 52.6]	< 0.001
**RDW**	14.4 (1.68)	15.7 (2.40)	< 0.001
INR*	1.20 [0.900, 4.10]	1.30 [0.900, 4.80]	< 0.001
PTT*	28.6 [17.0, 150]	31.1 [18.1, 150]	< 0.001
PT*	13.6 [10.0, 40.1]	14.5 [10.3, 49.9]	< 0.001
PEEP*	5.00 [0, 24.0]	5.00 [0, 24.0]	< 0.001
**Diabetes**			0.00337
Non-DM	583 (77.8%)	120 (67.0%)	
DM	166 (22.2%)	59 (33.0%)	
**UTI**			0.00105
Non-UTI	681 (90.9%)	147 (82.1%)	
UTI	68 (9.1%)	32 (17.9%)	
**Sepsis**			< 0.001
Non-Sepsis	699 (93.3%)	101 (56.4%)	
Sepsis	50 (6.7%)	78 (43.6%)	

* Wilcoxon rank-sum test

ARDS, acute respiratory distress syndrome; MIMIC, Medical Information Mort for Intensive Care; AKI, acute kidney injury; UTI, urinary tract infection; BMI, body mass index; BE, base excess; BUN, blood urea nitrogen; DBP, diastolic blood pressure; ABC, actual bicarbonate radical; HR, heart rate; MAP, mean arterial pressure; PCO_2_, partial pressure of carbon dioxide; PO_2_, partial arterial oxygen pressure; RBC, red blood cell; RR, respiratory rate; SBP, systolic blood pressure; SpO_2_, oxygen saturation; WBC, white blood cell; UO, urine output; ALT, alanine aminotransferase; AST, aspartate aminotransferase; TBIL, total bilirubin; RDW, red cell volume distribution width; INR, international normalized ratio; PTT, partial thromboplastin time; PT, prothrombin time; PEEP, positive end expiratory pressure

### Model development

**Fig. 2:**
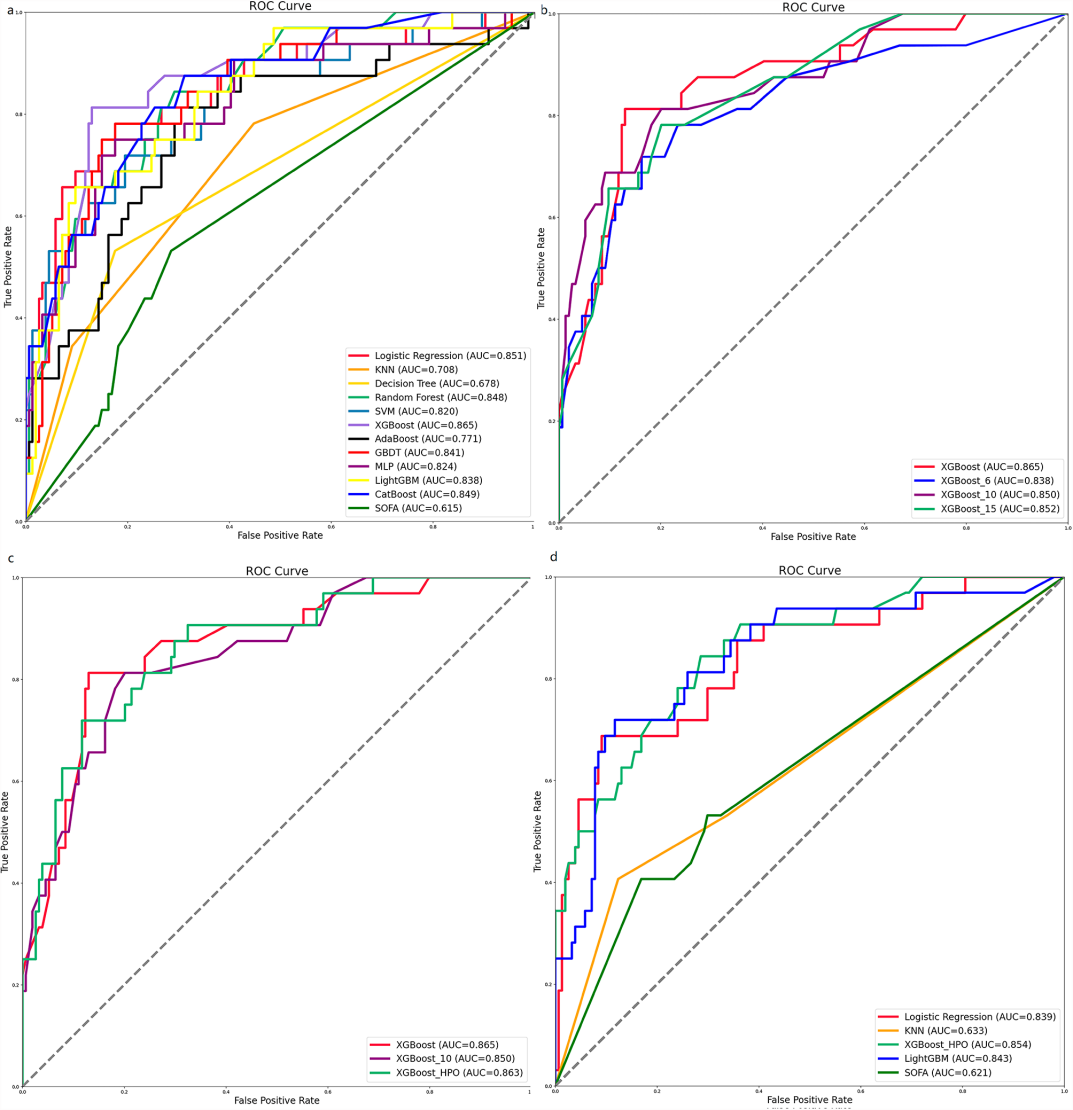
Receiver operating characteristic curves of ML models and SOFA score. ML, machine learning; KNN, K-nearest neighbor; SVM, support vector machine; XGBoost, eXtreme gradient boosting; AdaBoost, adaptive boosting; GBDT, gradient boosting decision tree; MLP, multi-layer perception; LightGBM, light gradients boosting machine; CatBoost, category boosting; HPO, hyperparameter optimization; SOFA, sequential organ failure assessment

**Fig. 3:**
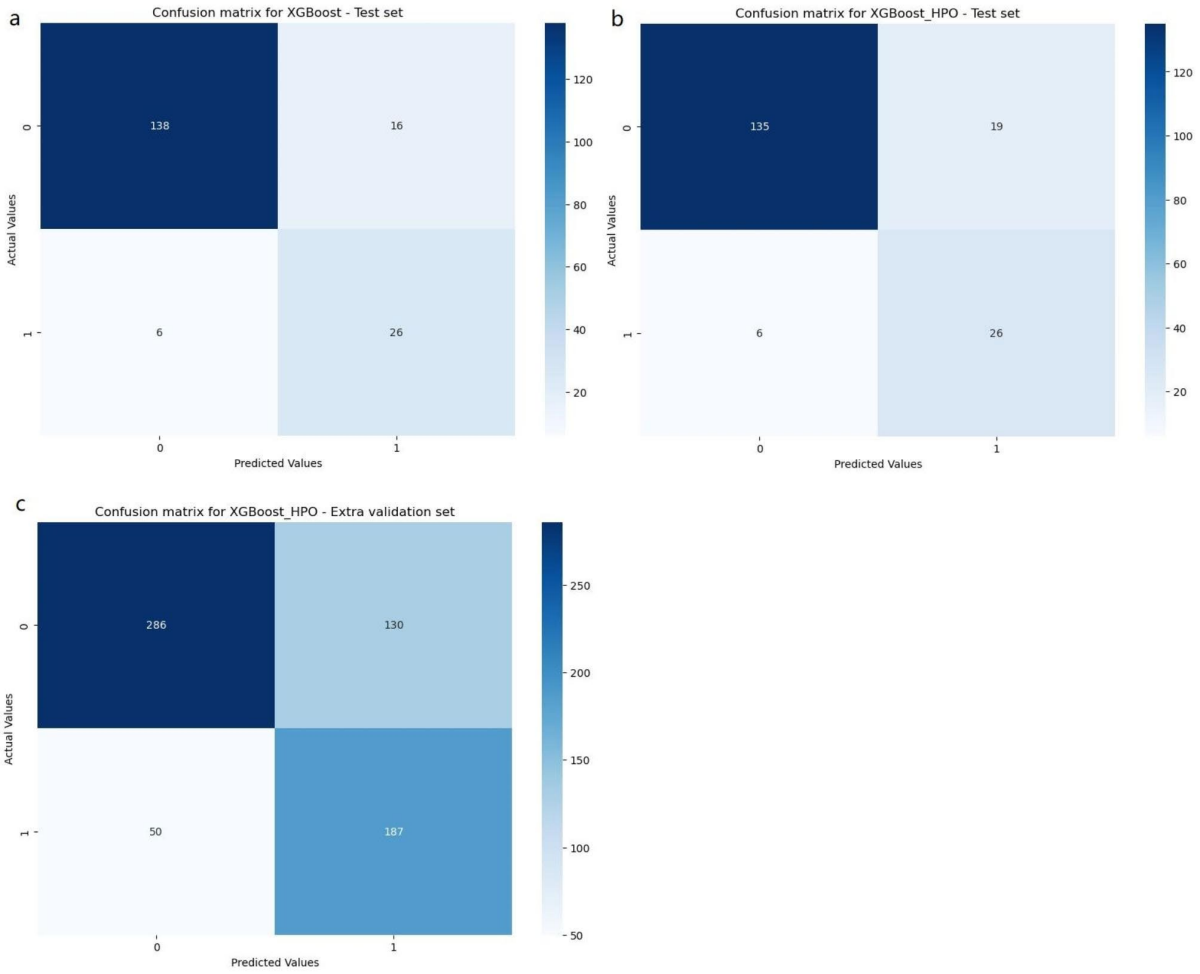
Confusion matrixs of XGBoost and XGBoost_HPO. **a** Confusion matrixs of XGBoost in testing set; **b** Confusion matrixs of XGBoost_HPO in testing set; **c** Confusion matrixs of XGBoost_HPO in extra validation set. XGBoost, eXtreme gradient boosting; HPO, hyperparameter optimization

We developed 11 ML binary classifiers using 742 cases from the training set, and used a testing set of 186 individuals to predict the risk of AKI in ARDS patients. The performance summary of the predictive models and the SOFA score on the testing set is presented in Table 2. It shows that the XGBoost model outperforms the other ML models and SOFA score in terms of accuracy (0.882), sensitivity(0.813), PPV (0.619), NPV (0.958), and F1 score (0.703). The XGBoost model has a specificity of 0.896, placing it at an intermediate level among the various models. Additionally, XGBoost provides relatively better model fitting performance, with the highest area under the curve (AUC) of 0.865 (Fig. 2a). Therefore, the XGBoost model was selected for further prediction. Fig. 3a illustrates the confusion matrix of the XGBoost model.

**Table 2:** Evaluation indicators in testing set of 11 ML models and SOFA score

Model	Accuracy	Sensitivity	Specificity	PPV	NPV	F1_score
Logistic Regression	0.823	0.688	0.851	0.489	0.929	0.571
KNN	0.731	0.500	0.779	0.320	0.882	0.390
Decision Tree	0.753	0.438	0.818	0.333	0.875	0.378
Random Forest	0.850	0.656	0.890	0.553	0.926	0.600
SVM	0.860	0.531	**0.929**	0.607	0.905	0.567
XGBoost	**0.882**	**0.813**	0.896	**0.619**	**0.958**	**0.703**
AdaBoost	0.780	0.375	0.864	0.364	0.869	0.369
GBDT	0.839	0.563	0.896	0.529	0.908	0.545
MLP	0.855	0.594	0.909	0.576	0.915	0.585
LightGBM	0.866	0.625	0.916	0.606	0.922	0.615
CatBoost	0.860	0.531	**0.929**	0.607	0.905	0.567
SOFA	0.699	0.500	0.740	0.286	0.877	0.364

ML, machine learning; KNN, K-nearest neighbor; SVM, support vector machine; XGBoost, eXtreme gradient boosting; AdaBoost, adaptive boosting; GBDT, gradient boosting decision tree; MLP, multi-layer perception; LightGBM, light gradients boosting machine; CatBoost, category boosting; PPV, positive prediction value; NPV, negative prediction value; SOFA, sequential organ failure assessment

### Feature importance analysis

**Fig. 4:**
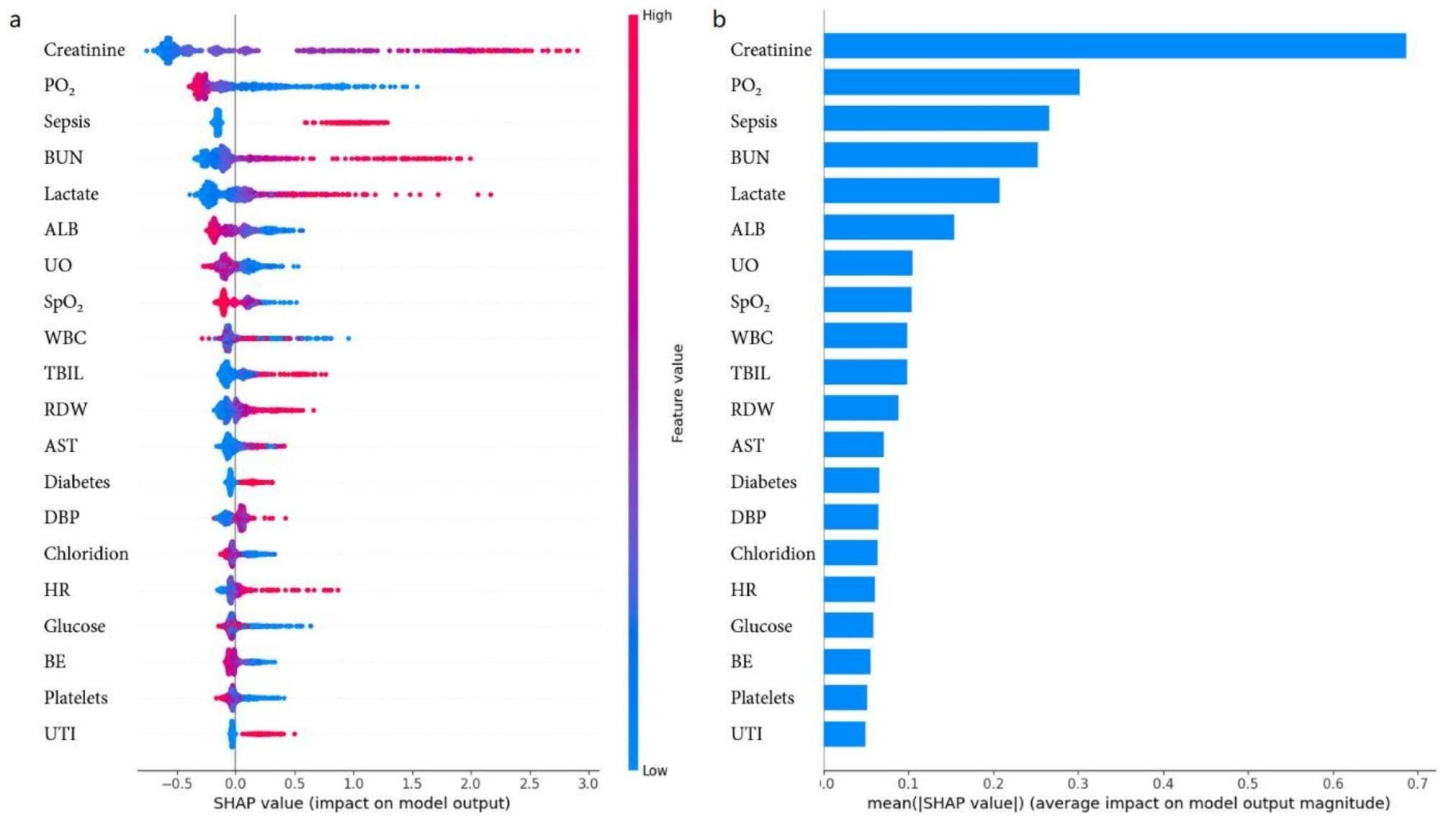
**a** SHAP values output by all patients in the XGBoost model; **b** The feature importance of XGBoost model. SHAP, shapley additive explanations; XGBoost, eXtreme gradient boosting; PO_2_, partial pressure of carbon dioxide; BUN, blood urea nitrogen; WBC, white blood cell; UO, urine output; ALB, albumin; AST, aspartate aminotransferase; SpO_2_, oxygen saturation; ALT, alanine aminotransferase; DBP, diastolic blood pressure; RBC, red blood cell; TBIL, total bilirubin; RDW, red cell volume distribution width; HR, heart rate; BE, base excess; UTI, urinary tract infection

Fig. 4 displays the distribution of the effects of each feature in the top 20 XGBoost model features, evaluated using SHAP value. Creatinine emerged as the most influential feature, followed by PO_2_, sepsis, BUN, lactate, ALB, UO, SpO_2_, WBC, TBIL, RDW, AST, diabetes, DBP, chloride, HR, glucose, BE, platelets, and UTI. These features were deemed critical in the XGBoost model.

### Model simplification and improvement

**Fig. 5:**
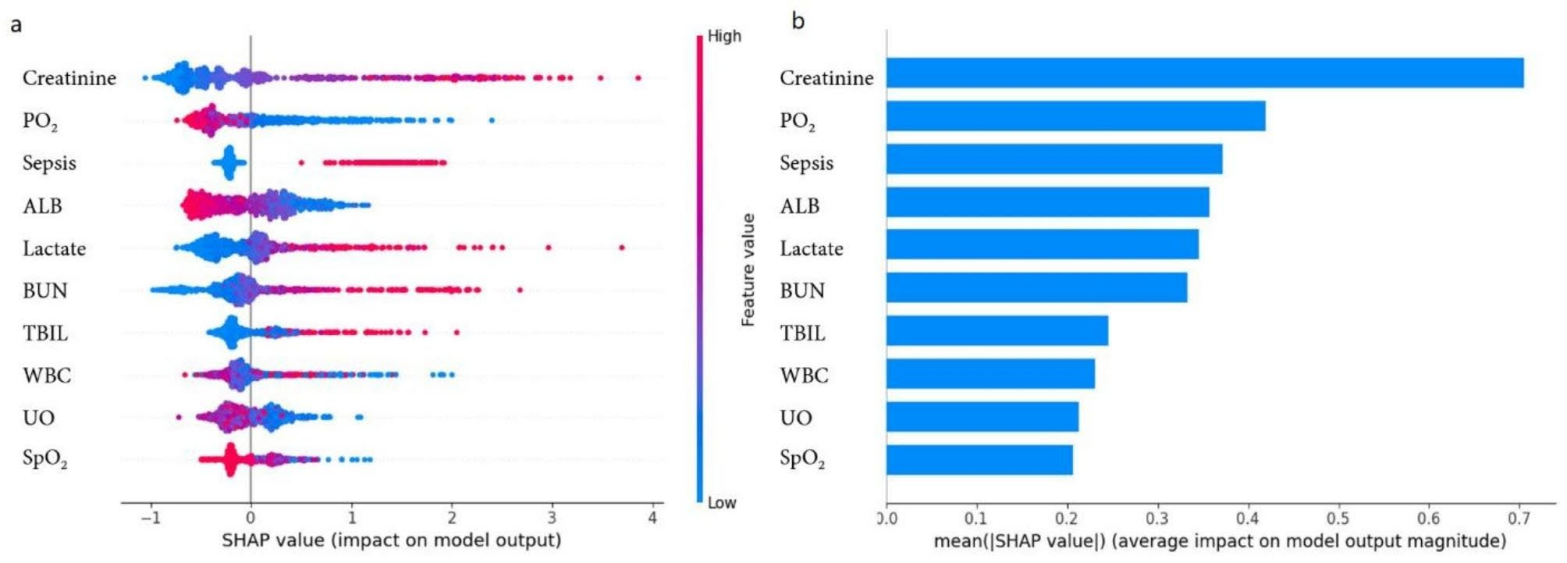
**a** SHAP values output by all patients in the XGBoost_10 model; **b** The feature importance of XGBoost_10 model. SHAP, shapley additive explanations; XGBoost, eXtreme gradient boosting; WBC, white blood cell; PO2, partial pressure of carbon dioxide; ALB, albumin; BUN, blood urea nitrogen; TBIL, total bilirubin;  UO, urine output; SpO_2_, oxygen saturation

To optimize the balance between model performance and clinical applicability, we developed 3 compact models using the top 15, top 10, and top 6 features. The compact model with 10 features achieved an AUC with 0.850 (Fig. 2b), indicating only a slight decrease in performance compared to the full model. Therefore, we selected the top 10 features for our final compact model. Fig. 5 demonstrates the importance of features in the compact model, which consists of 10 selected features. To enhance the performance of the compact model, we conducted HPO and obtained the XGBoost model with the best performance, as presented in Supplementary Fig. 1a. We have included the final settings of the hyperparameter search in Supplementary Table 1 and ranked the importance of various hyperparameters for model performance in Supplementary Fig. 1b. Supplementary Fig. 1c displays the performance of a single hyperparameter. A comparison was made between the 10-feature compact model with the optimal combination of model parameters and the pre-HPO model. As shown in Fig. 2c, the full model achieved an impressive AUC of 0.865, while the compact model had a slightly lower predictive performance with an AUC of 0.850. However, after applying HPO, the predictive value of the compact model improved as expected, resulting in an AUC of 0.863.

Other evaluation indicators including accuracy, sensitivity, specificity, PPV, NPV and F1 score of the different models based on the 10 features in the testing set are summarized in Table 3. It is apparent that, when compared to the XGBoost model, XGBoost_HPO model shows minimal decline across all evaluation metrics.

**Table 3:** Evaluation indicators in testing set of XGBoost, XGBoost_10 and XGBoost_HPO models

Model	Accuracy	Sensitivity	Specificity	PPV	NPV	F1_score
XGBoost	0.882	0.813	0.896	0.619	0.958	0.703
XGBoost_10	0.849	0.781	0.864	0.543	0.950	0.641
XGBoost_HPO	0.866	0.813	0.877	0.578	0.957	0.675

XGBoost, eXtreme gradient boosting; HPO, hyperparameter optimization; PPV, positive prediction value; NPV, negative prediction value

The data from the MIMIC-IV dataset was used to evaluate the performance of the XGBoost_HPO model. The study included a total of 635 individuals, and the mortality rate among them was 29%. The detailed characteristics of the patients can be found in Supplementary Table 2. ROC curves in testing set for XGBoost_HPO, KNN, logistic regression, LightGBM and SOFA are presented in Fig. 2d. Notably, the XGBoost_HPO model demonstrates the highest AUC (0.854) among them. The results in Table 4 indicate that the XGBoost_HPO model achieves the highest values for accuracy (0.724), specificity (0.688), PPV(0.590), and F1 score (0.675). However, in terms of sensitivity (0.789) and NPV (0.85), the XGBoost_HPO model ranks second and third.

**Table 4:** Evaluation indicators in extra validation set of XGBoost_HPO and other models

Model	Accuracy	Sensitivity	Specificity	PPV	NPV	F1_score
XGBoost	0.724	0.789	0.688	0.590	0.851	0.675
LightGBM	0.723	0.793	0.683	0.582	0.856	0.671
Logistic_ Regression	0.677	0.709	0.660	0.537	0.803	0.610
KNN	0.578	0.662	0.531	0.440	0.739	0.529
SOFA	0.699	0.531	0.734	0.293	0.883	0.378

KNN, K-nearest neighbor; XGBoost, eXtreme gradient boosting; HPO, hyperparameter optimization; LightGBM, light gradients boosting machine; PPV, positive prediction value; NPV, negative prediction value; SOFA, sequential organ failure assessment

**Fig. 6:**
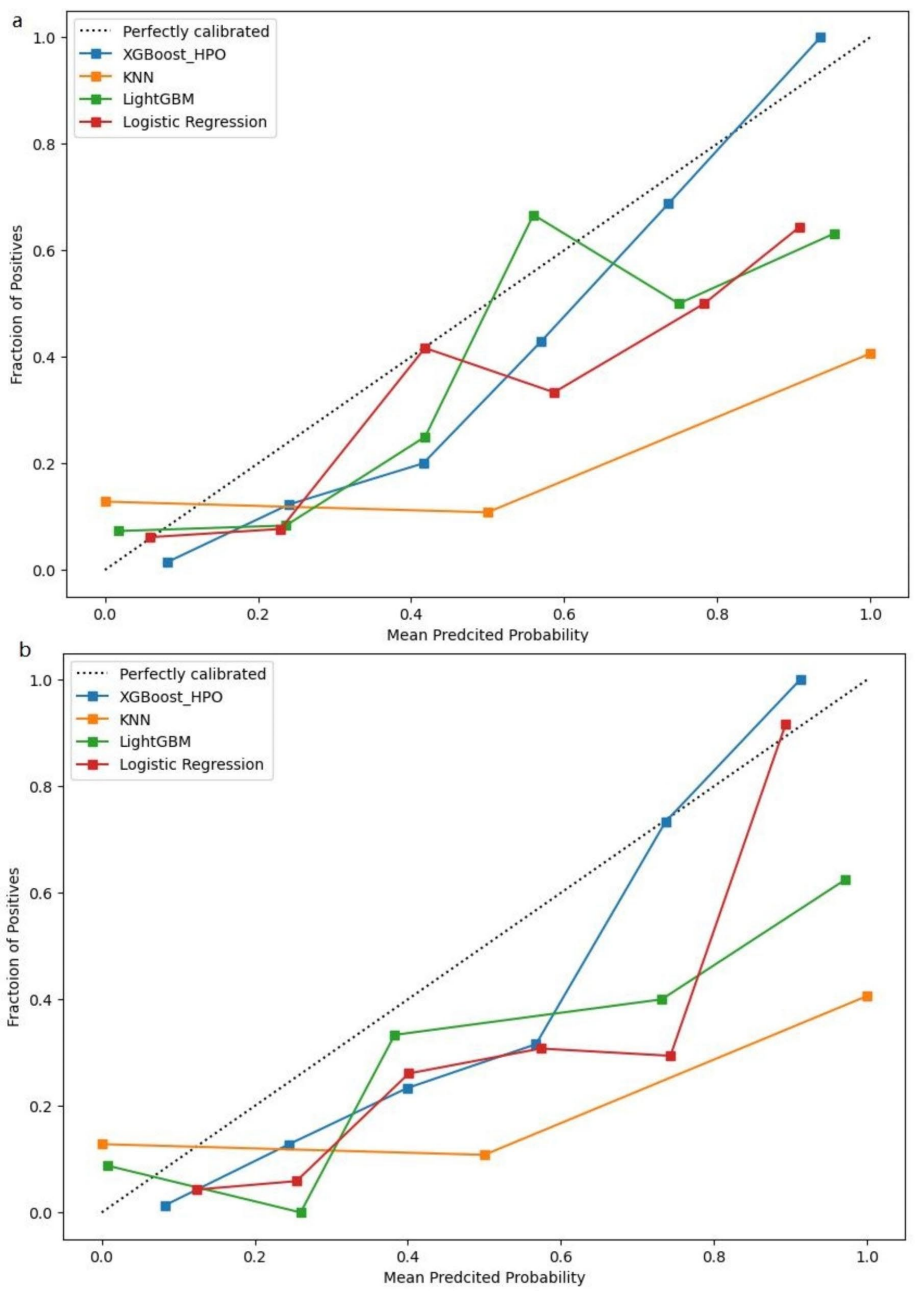
Calibration curves of logistic regression, LightGBM, KNN and XGBoost_HPO model. **a** Calibration curves in testing set; **b** Calibration curves in extra validation set. KNN, K-nearest neighbor; XGBoost, eXtreme gradient boosting; LightGBM, light gradients boosting machine; HPO, hyperparameter optimization

In this study, we conducted calibration curve plotting to evaluate the performance of various models. We compared the XGBoost_HPO model with logistic regression, LightGBM, and KNN models. Fig. 6 illustrates that the prediction probability of the XGBoost_HPO model. The XGBoost_HPO model exhibited superior calibration compared to the other models, both in the test and extra validation datasets. This further confirms the effectiveness of the XGBoost_HPO model in accurately predicting outcomes.

Finally, we developed a web-based interactive program using Gradio (a python framework that can demo a ML model easily for everyone to use), based on 10 features for predicting AKI and determining the probability (Supplementary Fig. 2). Supplementary Fig. 3 presents the decision curve analysis (DCA) curve related to the web calculator to determine the range of benefit for patients. The DCA curve shows that when the threshold probability for in-hospital AKI occurrence in patients is between 0.05 and 0.85, the application of XGBoost_HPO yields significantly higher net benefit compared to both the “Treat none” and “Treat all” strategies. This suggests that the model has good clinical utility. The main codes of this program were available at Hugging Face (https://huggingface.co/zysnathan/AKI-prediction/blob/main/aki_prediction.py).

## Discussion

ML has become increasingly popular in developing predictive models for various diseases [19–21]. In this study, we employed 11 ML algorithms to predict the probability of AKI in ARDS patients, utilizing the MIMIC-III database, and compared the results with the SOFA score. The study developed a highly effective and clinically accessible XGboost compact model with 10 features. Furthermore, the model’s performance is validated using the MIMIC-IV dataset.

In the 10 features of the simplified model, creatinine is the most valuable diagnostic tools for identifying AKI and remain the most significant features in our model. However, relying exclusively on creatinine to predict kidney injury has limitations [22]. Creatinine is a late indicator of kidney damage and can be influenced by various factors such as age, sex, diet, muscle mass, and medications [23]. UO is also one of the important features in the model. As one of the diagnostic criteria for AKI [24], UO shares similar characteristics with creatinine. It has a delayed response and lacks specificity [22]. BUN is a traditional biomarker utilized to evaluate renal function [25], although it lacks sensitivity and specificity in diagnosing AKI [26]. However, it is a prominent feature when it comes to predicting AKI. PO_2_ ranks second in terms of its importance in the model, which is not surprising. For a long time, hypoxia has been recognized as a significant factor in the pathogenesis of AKI. The combination of inadequate tissue oxygen supply and high oxygen demand is regarded as a primary factor that makes the kidney susceptible to acute ischemic injury [27]. This also explained why SpO_2_ plays an important role in ARDS models. Research has indicated that SpO_2_ holds significant importance in predicting the occurrence of acute kidney injury in patients with COVID-19 [28] and liver cirrhosis [29]. The infiltration of WBC into the injured kidneys via the circulatory system triggers the release of inflammatory mediators, including cytokines and chemical factors. These inflammatory substances contribute to kidney damage and exacerbate the injury [30, 31]. These infiltrating WBC play a crucial role in AKI. Serum albumin is a important factor for AKI. Albumin levels could be beneficial in identifying patients who are at a higher risk for AKI. There are various potential mechanisms that contribute to these effects, such as the expansion of intravascular volume, antioxidant properties, the preservation of renal perfusion, and glomerular filtration [32]. TBIL is one of the 10 important features, which may be associated with hepatorenal syndrome. When TBIL rises, the dilation of splanchnic vasculature and the intense increase in renal artery tone lead to renal cortex ischemia and hypoperfusion. This is one of the contributing factors that lead to the development of hepatorenal syndrome [33]. Sepsis also play an important role in the model. Research has demonstrated that the kidney is highly vulnerable to damage during sepsis. It is considered one of the organs most susceptible to injury. Additionally, around two-thirds of patients with septic shock experience AKI [34, 35].

ML algorithms have the ability to construct intricate models and generate precise predictions when provided with relevant features. When sufficient features is available, ML algorithms are expected to perform well [36]. In our study, we were able to achieve satisfactory ML performance despite utilizing a relatively small dataset consisting of only 928 patients. ML has long been proven to be a powerful tool for predicting the prognosis of ARDS. Huang et al. used random forest model to predict the in-hospital mortality rate, 30-day mortality rate, and 1-year mortality rate of ARDS patients, achieving AUC of 0.891, 0.883, and 0.892, respectively. Similarly, Rui Tang et al. utilized logistic regression model, XGBoost model, and artificial neural network model to predict in-hospital mortality rate in trauma-induced ARDS patients, achieving AUC of 0.737, 0.745, and 0.757, respectively [37]. These results indicate that ML has good predictive value for in-hospital mortality rate in ARDS patients caused by trauma. In addition, a study constructed a prognostic model for sepsis-induced ARDS patients using ML to predict the occurrence of AKI within 48 h of admission to the ICU, achieving a high AUC of 0.86 and accuracy of 0.81 [9]. Overall, these findings highlight the potential of ML algorithms to improve prognostic accuracy and guide clinical decision-making in ARDS. Our research also demonstrated the predictive capabilities of ML. While SOFA score is an essential component in critical care and is frequently used in various scenarios [38, 39], our study found that its ability to predict AKI occurrence in ARDS patients was relatively weak. With the availability of larger and more diverse datasets, the performance of ML models is expected to improve even further, offering clinicians valuable insights into the management of this challenging condition.

Notably, XGBoost outperforms other types of ML models in this study, including linear models. XGBoost is an improved gradient boosting algorithm that is particularly well-suited for low and medium dimensional data. In fact, XGBoost is frequently used to predict patient healthcare outcomes [40, 41]. In addition, our study resulted in the development of an online program, which is a valuable tool for physicians as it simplifies the process of identifying patients who are at a high risk of developing AKI.

Our study has some limitations. To begin with, Although validation has been performed in MMIC-IV database, further validation in additional cohorts is still needed to demonstrate its generalizability. Secondly, as an administrative database, there are certain inherent limitations that must be acknowledged. Some data may not be available. Thirdly, like all retrospective studies, there may be unmeasured confounding factors that could affect the results. These confounding variables may be difficult to account for in the study design, making it challenging to draw definite conclusions. Lastly, since the study is based on ICU patients, the findings cannot be generalized to other populations, such as non-ICU patients or healthy individuals. Therefore, caution must be taken while interpreting the results and applying them to other patient groups.

## Conclusion

ML models are reliable tools for predicting AKI in ARDS patients. Among all models, the XGBoost model demonstrates the best predictive performance, assisting clinical practitioners in identifying high-risk patients and implementing early interventions to improve prognosis. Additionally, the compact model and web-based calculator further enhance clinical usability. With the development of ML technology, it will have broader applications in the future medical field.

### Electronic supplementary material

Below is the link to the electronic supplementary material.

Supplementary Material 1

## Data Availability

The data that support the findings of this study are available from from MIT and BIDMC but restrictions apply to the availability of these data, which were used under license for the current study, and so are not publicly available. Data are however available from the corresponding author (Shubin Guo, Email: shubin007@yeah.net) upon reasonable request and with permission of Massachusetts Institute of Technology (MIT) and Beth Israel Deaconess Medical Center (BIDMC).
