# Supplementary material for: Machine learning-based prediction model of acute kidney injury in patients with acute respiratory distress syndrome
Source: BMC Pulm Med. 2023 Oct 3;23:370. doi: 10.1186/s12890-023-02663-6 (PMC10548692; doi:10.1186/s12890-023-02663-6)

Supplementary Table 1 Final settings of the hyperparameter search

| Hyperparameters | Best Values |
| --- | --- |
| n_estimators | 516 |
| learning_rate | 0.010199415682142065 |
| min_data_in_leaf | 7 |
| max_depth | 3 |
| num_leaves | 15 |
| colsample_bytree | 0.95 |
| subsample | 1.0 |
| reg_alpha | 0.09623819103594808 |

Supplementary Table 2 Baseline characteristics of ARDS patients in MIMIC-IV

|  | **Non-AKI (N=416)** | **AKI (N=237)** | **P-value** |
| --- | --- | --- | --- |
| **Creatinine** | 0.800 [0.200, 1.80] | 1.20 [0.200, 3.90] | <0.001 |
| **PO_2_** | 180 [39.0, 763] | 125 [39.0, 567] | <0.001 |
| **WBC** | 11.5 [0.100, 91.9] | 13.4 [0.100, 71.1] | 0.002 |
| **BUN** | 15.0 [3.00, 64.0] | 28.0 [7.00, 109] | <0.001 |
| **Albmuin** | 3.03 (0.649) | 2.77 (0.637) | <0.001 |
| **Lactate** | 1.70 [0.600, 10.7] | 2.10 [0.500, 19.6] | <0.001 |
| **UO** | 1760 [85.0, 12900] | 1260 [0, 11800] | <0.001 |
| **SpO_2_** | 98.3(4.67) | 96.2 (4.11) | <0.001 |
| **TBIL** | 0.600 [0.100, 39] | 0.800 [0.100, 50.7] | <0.001 |
| **Sepsis** |  |  |  |
| Non-Sepsis | 364 (87.5%) | 132 (55.7%) | <0.001 |
| Sepsis | 52 (12.5%) | 105 (44.3%) |  |

* Wilcoxon rank-sum test

AKI, acute kidney injury; BUN, blood urea nitrogen; PO2, partial arterial oxygen pressure; SpO2, oxygen saturation; WBC, white blood cell; UO, urine output; TBIL, total bilirubin

Supplementary 1 Hyperparameters optimization. a optimization history with different trials; b The importance of different hyperparameters; c The performance of single hyperparameter


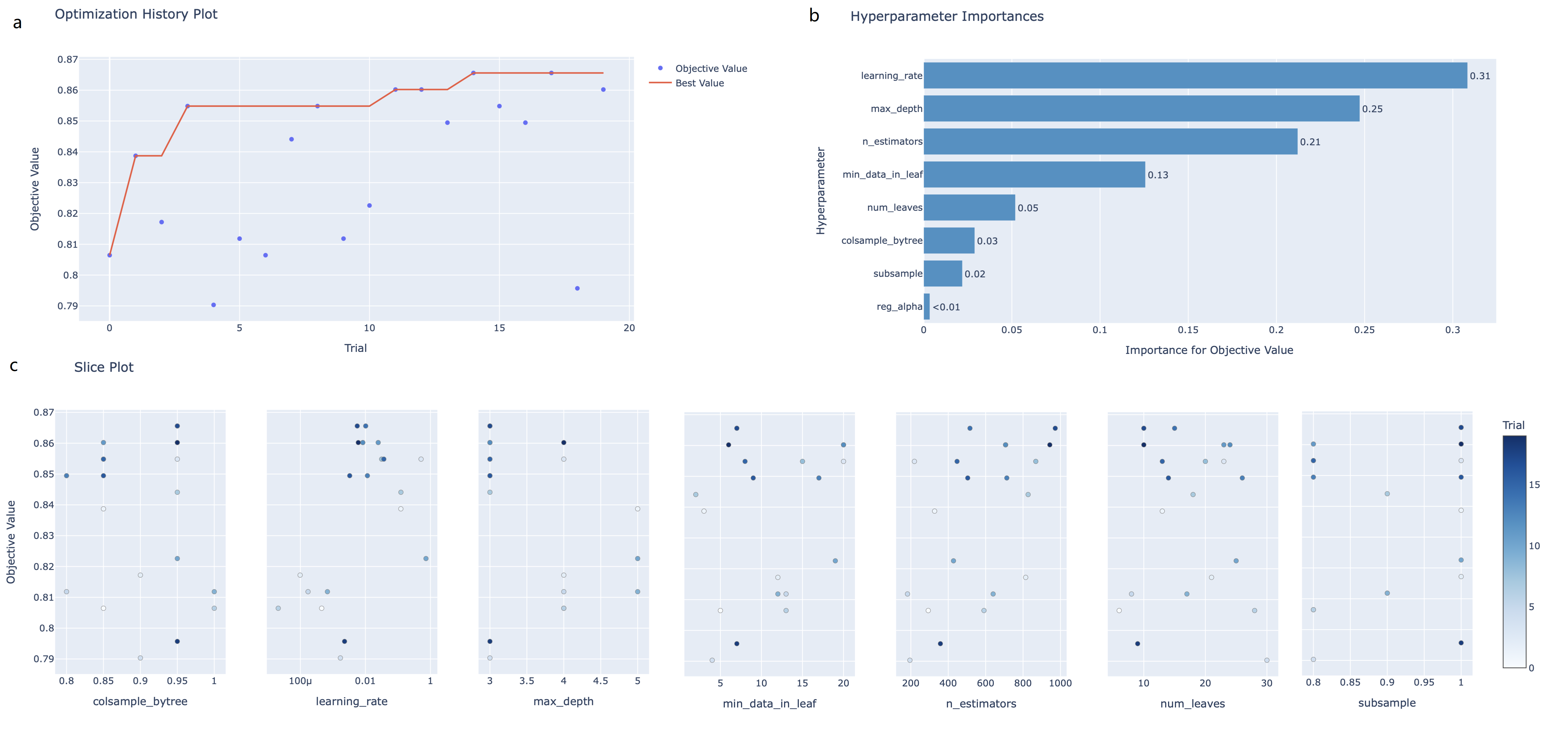


Supplementary 2 Operation interface of web-based interactive program


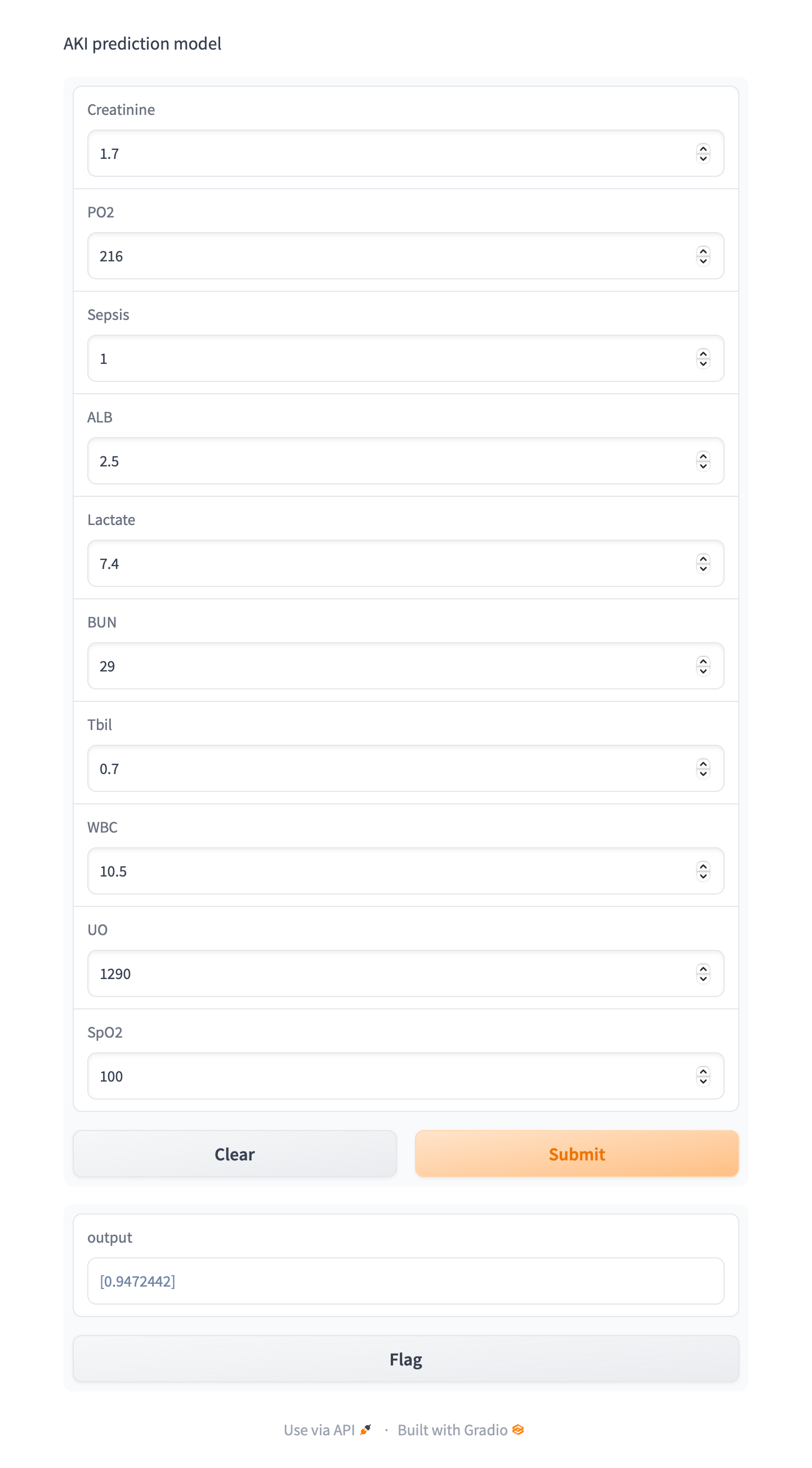


Supplementary Figure 3 DCA curve of XGBoostHPO model


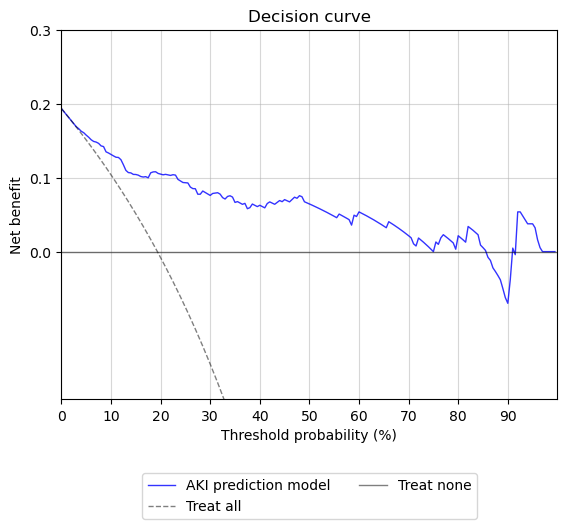

Supplement: Supplementary file 1 — Supplementary Material 1 [file 12890_2023_2663_MOESM1_ESM.docx]
